# Meta-analysis of diagnostic performance of serological tests for SARS-CoV-2 antibodies up to 25 April 2020 and public health implications

**DOI:** 10.2807/1560-7917.ES.2020.25.23.2000980

**Published:** 2020-06-11

**Authors:** Saverio Caini, Federica Bellerba, Federica Corso, Angélica Díaz-Basabe, Gioacchino Natoli, John Paget, Federica Facciotti, Simone Pietro De Angelis, Sara Raimondi, Domenico Palli, Luca Mazzarella, Pier Giuseppe Pelicci, Paolo Vineis, Sara Gandini

**Affiliations:** 1 Cancer Risk Factors and Lifestyle Epidemiology Unit, Institute for Cancer Research, Prevention and Clinical Network (ISPRO), Florence, Italy; 2 Department of Experimental Oncology, European Institute of Oncology (IEO), IRCCS, Milan, Italy; 3 Netherlands Institute for Health Services Research (NIVEL), Utrecht, the Netherlands; 4 Department of Hematology and Oncology, University of Milan, Milan, Italy; 5 MRC Centre for Environment and Health, School of Public Health, Imperial College, London, United Kingdom

**Keywords:** SARS-CoV-2, serological tests, sensitivity, specificity, predictive values, systematic review, meta-analysis

## Abstract

We reviewed the diagnostic accuracy of SARS-CoV-2 serological tests. Random-effects models yielded a summary sensitivity of 82% for IgM, and 85% for IgG and total antibodies. For specificity, the pooled estimate were 98% for IgM and 99% for IgG and total antibodies. In populations with ≤ 5% of seroconverted individuals, unless the assays have perfect (i.e. 100%) specificity, the positive predictive value would be ≤ 88%. Serological tests should be used for prevalence surveys only in hard-hit areas.

Testing of patients for ongoing infection with severe acute respiratory syndrome coronavirus 2 (SARS-CoV-2) is mostly conducted by detecting viral RNA in respiratory specimens using reverse transcription (RT)-PCR-based assays. While these tests can confirm infection, they may prove less helpful in quantifying the actual number of coronavirus disease (COVID-19) cases in the population, if a large proportion of infected individuals are either asymptomatic [1,2] or with mild symptoms, thereby having no incentive to seek medical care or to be tested. In this event, such cases may go unnoticed by surveillance systems and public health entities. Moreover, once the infection is resolved, RT-PCR tests do not inform on past infection. In order to overcome these shortcomings, serology-based tests are being increasingly used to gain more insight into the true prevalence of persons who have/have had COVID-19 and to assess the degree of herd immunity that has been acquired by the population. Serology-based tests have thus become a key public health element in the COVID-19 pandemic and there has been a rapid growth in the number of available SARS-CoV-2 serological tests since February 2020. These tests differ between one another in several ways, including the antigens used for antibody detection, the type of antibodies identified, and the laboratory method. Here, we conducted a systematic review and meta-analysis of the diagnostic accuracy of currently available SARS-CoV-2 serological tests, and assessed their real-world performance under scenarios of varying proportion of infected individuals in the population being tested.

## Searching studies assessing serological tests for severe acute respiratory syndrome coronavirus 2

We carried out a systematic literature search (up to 25 April 2020) of scientific articles on immunological tests for detection of SARS-CoV-2 antibodies. Both peer-reviewed and non-peer-reviewed reports in English were retrieved by interrogating the PubMed, medRxiv and bioRxiv databases with the following keywords: ‘SARS-COV-2 OR COVID’ AND ‘IgM OR IgG OR IgA OR antibody OR serological’ AND ‘test’. The search also extended to the reference lists of the reports found and to technical manuals of tests mentioned therein. Reports/technical documents considered in this review are referred to as ‘studies’ henceforth. We considered independent studies that specified the antigen used for antibody detection, used quantitative methods, and reported the number of true positives, true negatives, false positives, and false negatives. This information was extracted from each study as well as the laboratory method used as reference.

## Calculating performance parameters of the tests used in the studies

Based on the 2×2 contingency table, we calculated the test sensitivity and specificity (with 95% confidence intervals (CI)) and the diagnostic odds ratio (DOR), to provide an overall measure of the test performance [3]. We then calculated the positive (PPV) and negative (NPV) predictive values assuming a true prevalence of 5%, 10% and 20%. 

## Estimation of performance parameters overall

Concerning the pooled estimates of the performance parameters of serological tests, it should be noted that some of the studies included in the current systematic review assessed more than one assay. Among three investigations employing the ‘Beijing Wantai’ kit (Beijing Wantai Biological, Beijing, China), one also used the ‘Xiamen InnoDx Biotech’ kit (Xiamen InnoDx Biotech Co., Xiamen, China) to measure in particular IgG and total antibodies [4]. In the meta-analysis for calculating summary values, we only entered data for the ‘Beijing Wantai’ kit (instead of the ‘Xiamen InnoDx Biotech’ kit) from this study [4]. This was for consistency with the other studies found. Moreover, when tests with the nucleocapsid (N) protein and the spike protein antigens were both reported in a single study, we only entered data derived from assays with the N protein, because they generally showed better sensitivity. To assess the robustness of this choice, sensitivity analyses were conducted, whereby parameters were re-calculated by swapping data obtained from assays based on the N protein with those obtained based on the spike protein.

Pooled estimates of sensitivity and specificity were obtained through random-effects models after Freeman–Tukey double arcsine transformation. DOR were pooled by fitting a bivariate model, which takes into account the correlation between sensitivity and specificity and uses their log-transformed values as normally distributed variables.

Between-studies heterogeneity was assessed using the I^2^ statistics, which quantifies the percentage of variation attributable to heterogeneity rather than chance. An I^2^ below 50% was considered as an indicator of acceptable heterogeneity.

## Description of studies included in the systematic review

Following the identification of 71 records, screening led to the exclusion of 61 of these, leaving nine studies included in the current systematic review [4-12] (Figure). 

**Figure:**
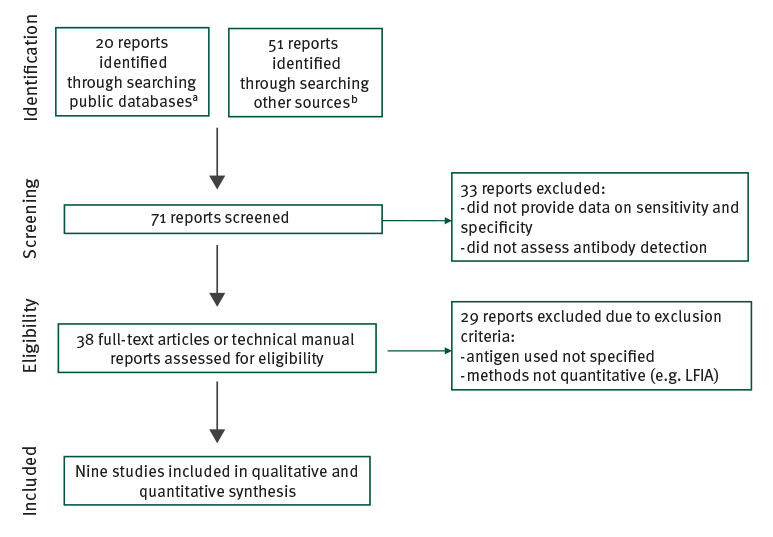
Flow-chart of the literature search up to 25 April 2020, of the diagnostic performance of serological tests for severe acute respiratory syndrome coronavirus 2 antibodies

LFIA: lateral flow immunoassay***.***

^a ^Including PubMed, medRxiv and bioRxiv databases.

^b^ Including reference lists of reports found through public databases and technical manuals of serological tests mentioned in these reports.

Six of the nine studies were based on commercial assays including enzyme-linked immunosorbent assay (ELISA) or chemiluminescence microparticle immunoassay (CMIA)/chemiluminescent immunoassay (CLIA), and three on in-house tests, for detecting SARS-CoV-2 antibodies (Table 1 and Supplementary Table S1). Most studies (n = 8) evaluated sensitivity and specificity separately for IgG and IgM, while only some (n = 4) reported those values for total antibodies. Only one study tested IgA [10]. Real-time RT-PCR was always used as the reference method for sensitivity, while the definition of patients testing negative varied across studies (Supplementary Table S2).

**Table 1:** Main characteristics of studies included in the systematic review and meta-analysis of the performance serological tests for SARS-CoV-2, along with test sensitivity and specificity and positive and negative predictive values assuming a true COVID-19 prevalence of 5%, 10% and 20% in the population tested, as at 25 April 2020 (n = 9 studies)

Study/report	Method	Ab detected	Number of patients	Sens	Spec	PPV-5^a^	PPV-10^a^	PPV-20^a^	NPV-5^b^	NPV-10^b^	NPV-20^b^	Test used	Antigen for detection
Lou et al. [4]	Beijing Wantai kit^c^	Total Ab	180	0.98	1.00	1.00	1.00	1.00	1.00	1.00	0.99	ELISA	RBD
	Xiamen InnoDx Biotech kit^c^	Total Ab	180	0.96	0.99	0.88	0.94	0.97	1.00	1.00	0.99	CMIA	RBD
	Beijing Wantai kit^c^	IgM	380	0.93	1.00	1.00	1.00	1.00	1.00	0.99	0.98	ELISA	RBD
	Xiamen InnoDx Biotech kit^c^	IgM	380	0.86	0.99	0.87	0.93	0.97	0.99	0.98	0.97	CMIA	RBD
	Beijing Wantai kit^c^	IgG	180	0.89	1.00	1.00	1.00	1.00	0.99	0.99	0.97	ELISA	N protein
Lin et al. [5]	In house protocol	IgG	159	0.82	0.98	0.63	0.79	0.89	0.99	0.98	0.96	CLIA	N protein
		IgM	159	0.82	0.81	0.19	0.33	0.52	0.99	0.98	0.95	CLIA	N protein
Liu et al. [6]	In house protocol	IgG	314	0.74	1.00	1.00	1.00	1.00	0.99	0.97	0.94	ELISA	Spike protein
		IgM	314	0.77	1.00	1.00	1.00	1.00	0.99	0.98	0.95	ELISA	Spike protein
		IgG	314	0.70	1.00	1.00	1.00	1.00	0.98	0.97	0.93	ELISA	N protein
		IgM	314	0.68	1.00	1.00	1.00	1.00	0.98	0.97	0.93	ELISA	N protein
Zhao et al. [7]	Beijing Wantai kit^c^	Total Ab	386	0.93	0.99	0.84	0.92	0.96	1.00	0.99	0.98	ELISA	RBD
		IgG	370	0.65	0.99	0.77	0.88	0.94	0.98	0.96	0.92	ELISA	N protein
		IgM	386	0.83	0.99	0.76	0.87	0.94	0.99	0.98	0.96	ELISA	RBD
Creative Diagnostics [8]	Kit	IgG	46	1.00	1.00	1.00	1.00	1.00	1.00	1.00	1.00	ELISA	Whole virus lysate
Epitope Diagnostic [9]	Kit	IgG	84	1.00	1.00	1.00	1.00	1.00	1.00	1.00	1.00	ELISA	N protein
Lassaunière et al. [10]	Beijing Wantai kit^c^	Total Ab	112	0.93	1.00	1.00	1.00	1.00	1.00	0.99	0.98	ELISA	RBD
	Euroimmun kit^c^	IgA	112	0.93	0.93	0.41	0.60	0.77	1.00	0.99	0.98	ELISA	RBD
		IgG	112	0.67	0.96	0.47	0.65	0.81	0.98	0.96	0.92	ELISA	RBD
Ortho-Clinical Diagnostics [11]	Kit^c^	Total Ab	436	0.83	1.00	1.00	1.00	1.00	0.99	0.98	0.96	ELISA	Spike protein
Adams et al. [12]	In house protocol	IgG	90	0.85	1.00	1.00	1.00	1.00	0.99	0.98	0.96	ELISA	Spike protein
		IgM	90	0.70	1.00	1.00	1.00	1.00	0.98	0.97	0.93	ELISA	Spike protein

Ab: antibody; CLIA: chemiluminescent immunoassay; CMIA: chemiluminescent microparticle immunoassay; COVID-19: coronavirus disease; ELISA: enzyme-linked immunosorbent assay; NPV: negative predictive value; PPV: positive predictive value; RBD: receptor-binding domain; SARS-CoV-2: severe acute respiratory syndrome coronavirus 2; sens: sensitivity; spec: specificity.

^a ^PPV-5, PPV-10, and PPV-20 are PPVs at a hypothesised prevalence of 5%, 10% and 20%.

^b ^NPV-5, NPV-10, and NPV-20 are NPVs at a hypothesised prevalence of 5%, 10% and 20%.

^c ^Commercial kit.

## Performances of serological assays investigated in individual studies

The reviewed studies had sample sizes ranging between 46 and 436 patients. For IgM, sensitivity ranged from 68% in Liu et al. (in-house test) [6] to 93% in Lou et al. (Beijing Wantai kit) [4], based on 314 and 380 patients, respectively. The lowest sensitivity for IgG detection (65%) was reported in Zhao et al. (Bejiing Wantai kit) [7], which tested 370 patients, while two smaller-size studies (46 and 84 patients) reached a sensitivity of 100% [8,9].

For IgM testing, the PPV had lowest values of 19% to 52% (in the 5% and 20% true-prevalence scenarios, respectively) in the study by Lin et al. (n = 159 patients) [5], while it was 100% in all scenarios in another study [12] and in the largest study (n = 314 patients) by Liu et al. [6]. For IgG, the PPV ranged between 47% and 81% (depending on the assumed prevalence) in the study by Lassaunière et al. (n = 112 patients) [10], while it amounted to 100% in other studies [4,6,8,9,12] as well as in the one by Lou et al. with the largest number of patients (n = 380) [4]. The NPV fell in the range 96–100% for all IgG and IgM kits when the prevalence was assumed to be 10% (the lower limit of the range became 98% and 92% for the 5% and 20% true-prevalence scenarios, respectively) as shown in Table 1.

## Synthesis of study results

Meta-analysis yielded a summary sensitivity of 82% (95%CI: 75–88%) for IgM, and 85% for both IgG (95%CI: 73–93%) and total antibodies (95%CI: 74–94%) (Table 2 and Supplementary Figure S1–S6). Pooled specificity was 98% (95%CI: 92–100%) for IgM and 99% (95%CI: 98–100%) for both IgG and total antibodies. Mostly due to the low proportion of false positives, the pooled DOR was generally very high (ca 2,800 for IgM, and ca 1,300 for IgG and total antibodies). Both sensitivity and specificity were 93% for the only assay testing IgA.

**Table 2:** Summary estimates of sensitivity and specificity, with 95% confidence intervals, of the serological tests for SARS-CoV-2 included this systematic review, as at 25 April 2020

Parameter	Summary estimate (95%CI); I^2^		
	IgM	IgG	Total Ab
Sensitivity	0.82 (0.75–0.88); I^2^ = 72%	0.85 (0.73–0.93); I^2^ = 88%	0.85 (0.74–0.94); I^2^ = 79%
Specificity	0.98 (0.92–1.00); I^2^ = 92%	0.99 (0.98–1.00); I^2^ = 13%	0.99 (0.98–1.00); I^2^ = 74%

SARS-CoV-2: severe acute respiratory syndrome coronavirus 2.

Results remained substantially unaltered in all sensitivity analyses (data not shown).

Heterogeneity was consistently high (I^2^ > 50%), except when pooling specificity of IgG tests (I^2^ = 13%).

## Discussion

While some SARS-CoV-2 serological tests reported an excellent ability to discriminate between seroconverted and non-seroconverted individuals, others showed markedly lower diagnostic accuracy. In particular, the pooled sensitivity for all/all types of antibodies was unsatisfactory (82–85%), as a substantial fraction (one sixth on average) of seroconverted individuals would be incorrectly classified as non-seroconverted. Specificity was generally very high (≥ 98%), yet this may not suffice to guarantee satisfactory real-world performance in areas with a very low prevalence of infected individuals. A specificity just less than perfect (99%) would in fact produce a PPV ranging between 76% and 88% when combined with a true prevalence equal to 5%, meaning that around one fifth of those labelled as seroconverted would in reality be false positives.

According to the World Health Organization (WHO), 2–3% of the global population may have been infected by the end of the first epidemic wave [13], thus the PPV in most areas could indeed be much lower than in our simulations. Further reasons of concern lie in the low number of patients on whom some estimates are based, the variability in terms of the gold standard used to define sensitivity and specificity, the possible heterogeneity of testing procedures (which should be harmonised internationally to ensure comparability), the fact that some of the included studies were not, or had not yet been peer-reviewed [4,5,8-12], and, above all, the uncertainty as to whether positivity according to the test means that effective protection against re-infection has been established [14,15]. Of note, some of the above factors may have contributed to widen the range of reported of sensitivity and specificity between studies and, in turn, to the high heterogeneity observed in most of the pooled results. Our choice to consider the N protein instead of the spike protein was justified by the generally better sensitivity for the former, but can be questioned as the latter is generally more likely to induce neutralising antibodies. 

While the available serological tests included here can be used for research purposes, our data suggest that their use for large-scale prevalence surveys (or to grant ‘immunity passports’, which could possibly entail exemption from use of personal protective equipment for healthcare personnel, and face masks and social distancing measures for the general population) appears currently only justified (and only if showing very high diagnostic accuracy) in hard-hit regions, while they should be used with caution elsewhere. Moreover, issues of cost, speed, and availability should also be taken into account when planning large seroprevalence surveys, as well as the medical and non-medical costs of diagnostic errors. Finally, SARS-CoV-2 serological tests are being developed at a fast pace, and the conclusions of our report may need revision in the coming months, also depending on the further spread of the pandemic. 

## Supplementary Data

137000 Supplementary Material
