# Supplementary material for: Meta-analysis of diagnostic performance of serological tests for SARS-CoV-2 antibodies up to 25 April 2020 and public health implications
Source: Euro Surveill. 2020 Jun 11;25(23):2000980. doi: 10.2807/1560-7917.ES.2020.25.23.2000980 (PMC7403641; doi:10.2807/1560-7917.ES.2020.25.23.2000980)

This supplementary material is hosted by *Eurosurveillance* as supporting information alongside the article **Meta-analysis of diagnostic performance of serological tests for SARS-CoV-2 antibodies up to 25 April 2020 and public health implications**, on behalf of the authors, who remain responsible for the accuracy and appropriateness of the content. The same standards for ethics, copyright, attributions and permissions as for the article apply. Supplements are not edited by *Eurosurveillance* and the journal is not responsible for the maintenance of any links or email addresses provided therein.

Supplementary Table S1. Methods of reference to identify true positives and true negatives.

Test name	Identification of positive subjects	Identification of negative subjects
Lou et al. [4]	SARS-CoV-2 through real-time RT-PCR (rRT-PCR) testing.	SARS-CoV-2 through real-time RT-PCR (rRT-PCR) testing.
Lin et al [5]	Combinations of epidemiological risk factors, clinical features and positive detections of SARS-CoV-2 RNA in respiratory specimens	Combination of epidemiological risks, and persistently negative for SARS-CoV-2 RNA detections in at least three respiratory specimens' tests.
Liu et al. [6]	SARS-CoV-2 through real-time RT-PCR (rRT-PCR) testing at a median of 15 d.p.o. (range. 0–55 days).	Serum samples from 100 healthy blood donors.
Zhao et al. [7]	SARS-CoV-2 by use of real-time RT-PCR (rRT-PCR)	Samples collected from healthy individuals before the outbreak of SARS-CoV-2.
Creative Diagnostics [8]	SARS-CoV-2 by use of real-time RT-PCR (rRT-PCR) after the second week of the onset of the disease.	Normal healthy patients with samples collected prior to the SARS-COV-2 outbreak.
Epitope Diagnostic [9]	SARS-CoV-2 by use of real-time RT-PCR (rRT-PCR) after the second week of the onset of the disease.	Normal healthy patients with samples collected prior to the COVID outbreak.
Lassaunière et al. [10]	SARS-CoV-2 by use of real-time RT-PCR (rRT-PCR)	Samples collected from healthy individuals before the outbreak of SARS-CoV-2.
Ortho-Clinical Diagnostics [11]	SARS-CoV-2 by use of real-time RT-PCR (rRT-PCR)	400 presumed SARS-CoV-2 negative samples from healthy blood donors were tested.
Adams et al. [12]	SARS-CoV-2 through real-time RT-PCR (rRT-PCR)	Plasma samples collected from healthy individuals before the outbreak of SARS-CoV-2.

Supplementary Figure S1. Forest plot of the sensitivity of serological test for the detection of anti-SARS-CoV-2 IgM.

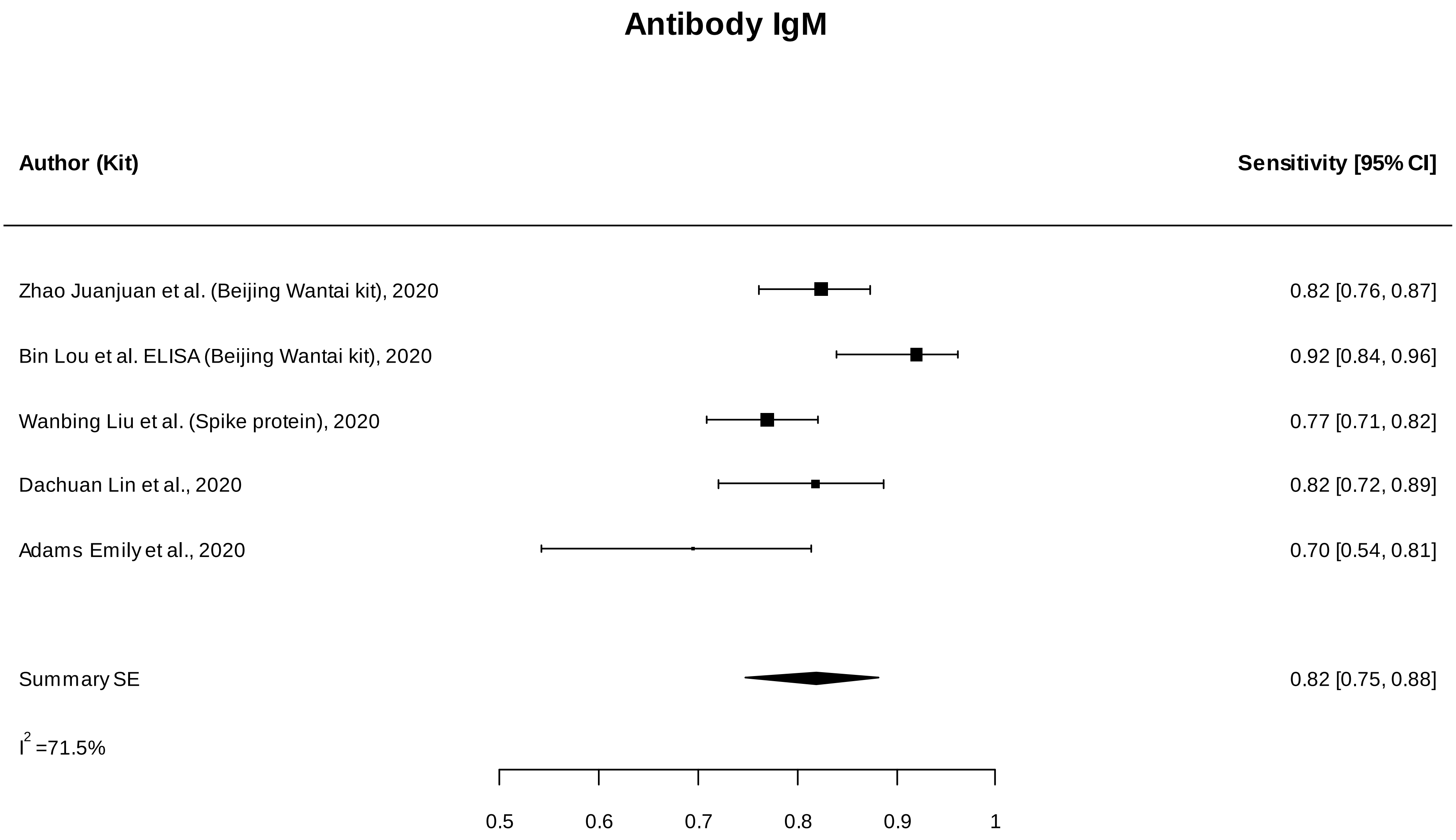

Supplementary Figure S2. Forest plot of the sensitivity of serological test for the detection of anti-SARS-CoV-2 IgG.

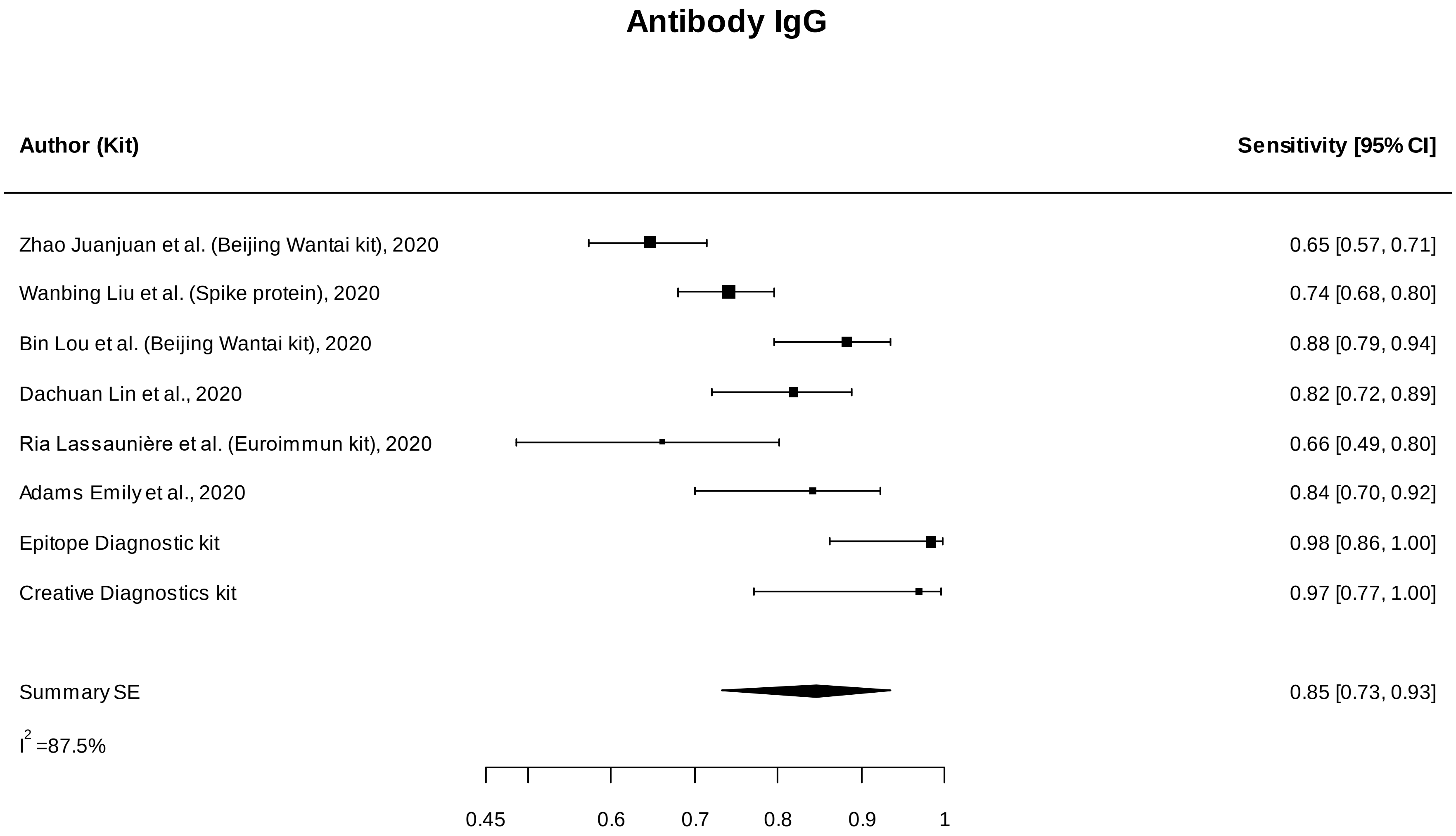

Supplementary Figure S3. Forest plot of the sensitivity of serological test for the detection of anti-SARS-CoV-2 total antibodies.

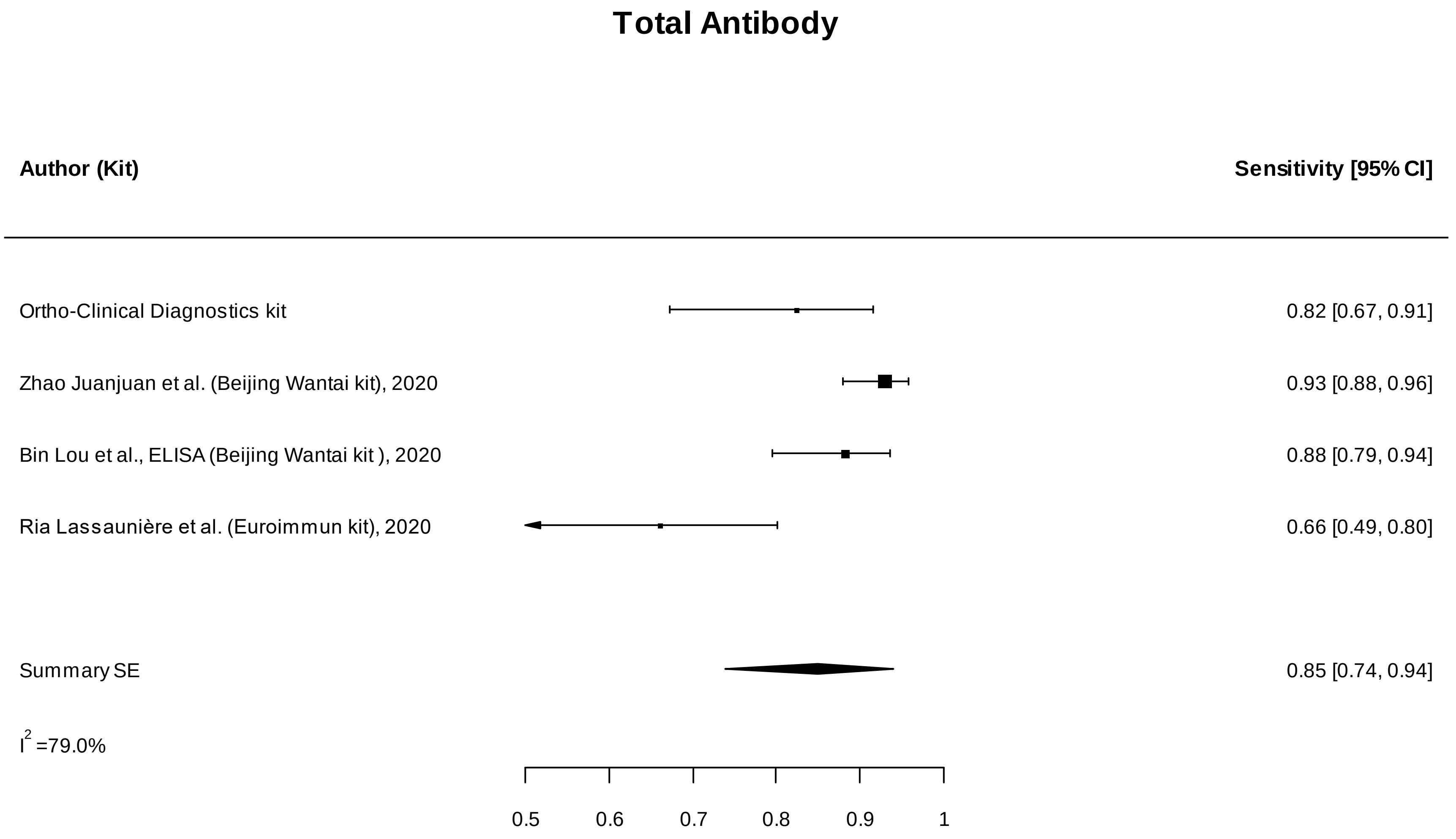

Supplementary Figure S4. Forest plot of the specificity of serological test for the detection of anti-SARS-CoV-2 IgM.

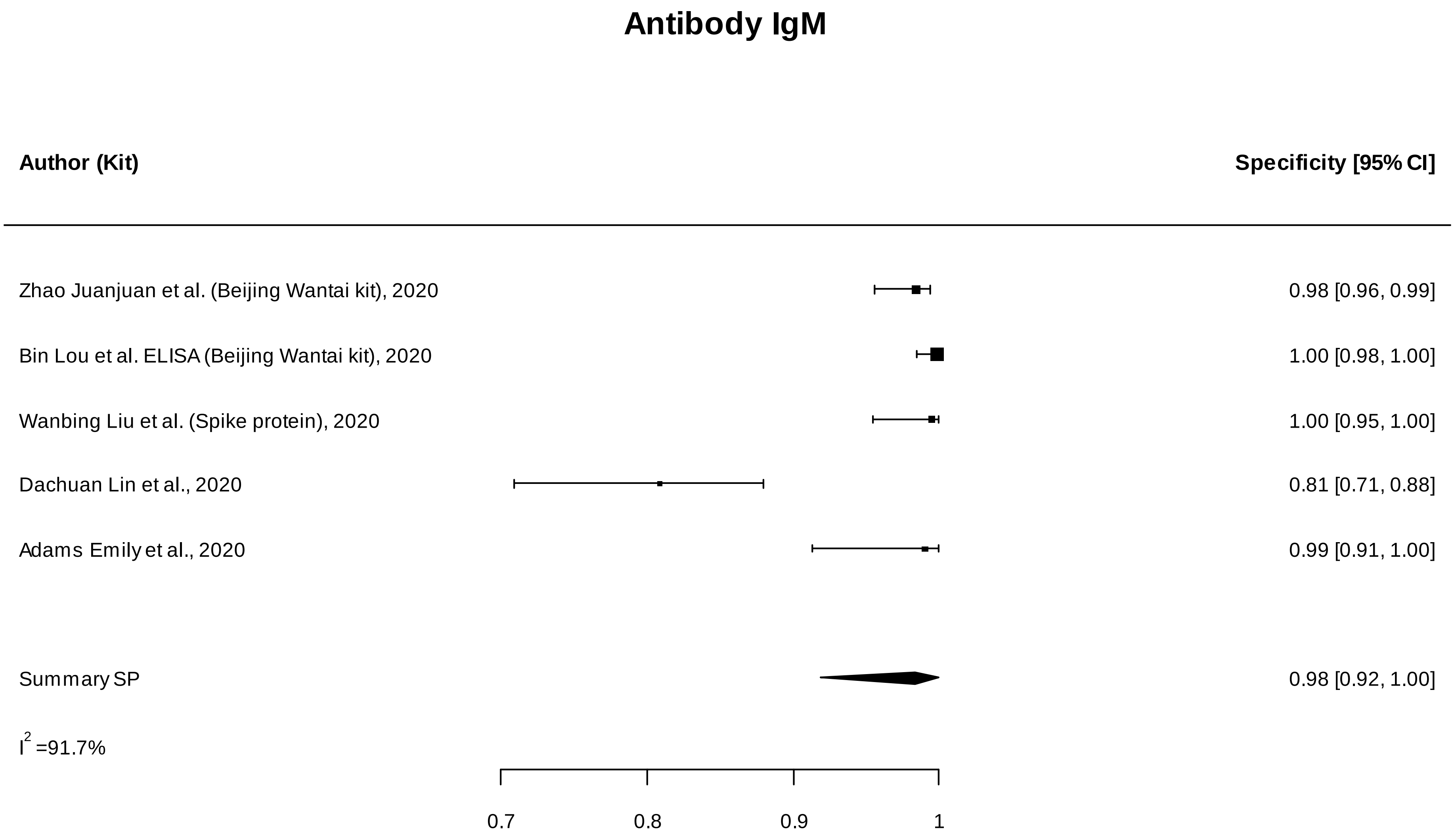

Supplementary Figure S5. Forest plot of the specificity of serological test for the detection of anti-SARS-CoV-2 IgG.

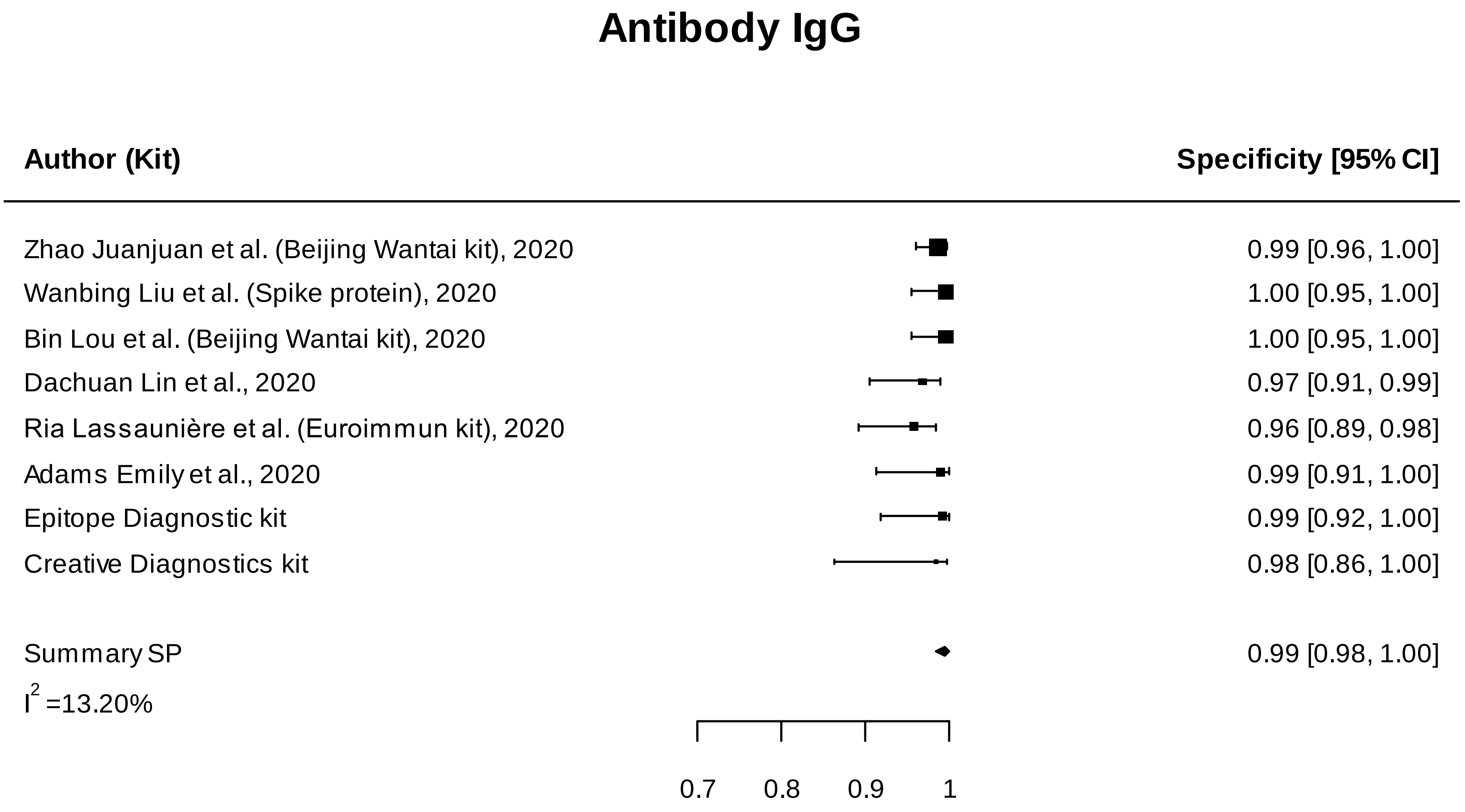

Supplementary Figure S6. Forest plot of the specificity of serological test for the detection of anti-SARS-CoV-2 total antibodies.

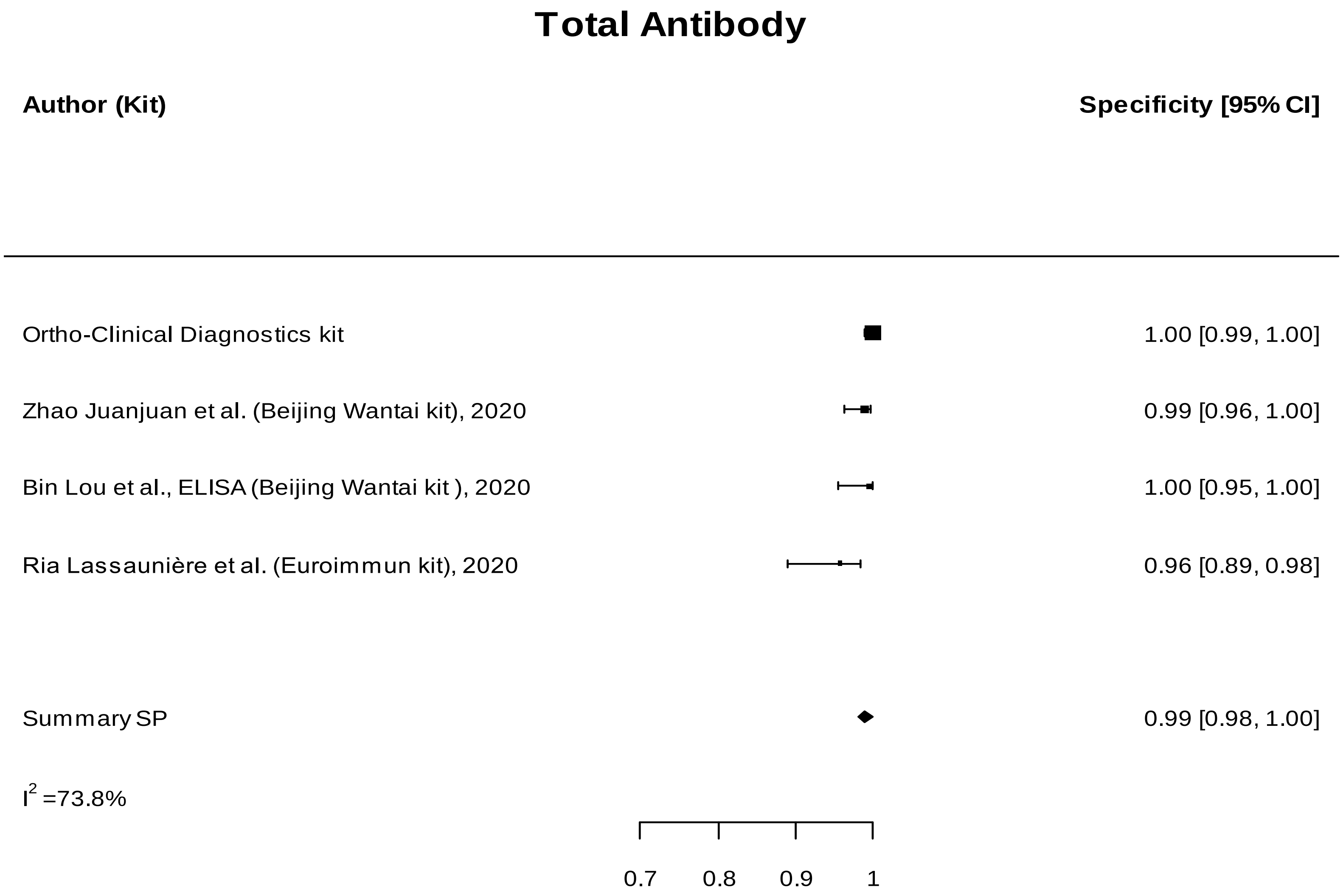

Supplement: Supplementary Material [file 2000980_CAINI_Supplementary_Material.pdf]
